# Association of Attention-Deficit/Hyperactivity Disorder With Teenage Birth Among Women and Girls in Sweden

**DOI:** 10.1001/jamanetworkopen.2019.12463

**Published:** 2019-10-02

**Authors:** Charlotte Skoglund, Helena Kopp Kallner, Alkistis Skalkidou, Anna-Karin Wikström, Cecilia Lundin, Susanne Hesselman, Anna Wikman, Inger Sundström Poromaa

**Affiliations:** 1 Department of Clinical Neuroscience, Karolinska Institute, Stockholm, Sweden; 2 Danderyd Hospital, Department of Clinical Sciences, Karolinska Institute, Stockholm, Sweden; 3 Department of Department of Women’s and Children’s Health, Uppsala University, Uppsala, Sweden; 4 Center for Clinical Research, Uppsala University, Falun, Sweden

## Abstract

**Importance:**

Attention-deficit/hyperactivity disorder (ADHD) is associated with a plethora of adverse health outcomes throughout life. While Swedish specialized youth clinics have carefully and successfully targeted risk of unplanned pregnancies in adolescents, important risk groups, such as women and girls with ADHD, might not be identified or appropriately assisted by these interventions.

**Objectives:**

To determine whether women and girls with ADHD are associated with increased risk of teenage birth compared with their unaffected peers and to examine the association of ADHD with risk factors for adverse obstetric and perinatal outcomes, such as smoking, underweight or overweight, and substance use disorder.

**Design, Setting, and Participants:**

This nationwide cohort study included data from 6 national longitudinal population-based registries in Sweden. All nulliparous women and girls who gave birth in Sweden between January 1, 2007, and December 31, 2014, were included. Data analyses were conducted from October 7, 2018, to February 8, 2019.

**Exposures:**

Women and girls treated with stimulant or nonstimulant medication for ADHD (Anatomic Therapeutic Chemical classification code N06BA) in the Swedish Prescribed Drug Register between July 1, 2005, and December 31, 2014.

**Main Outcomes and Measures:**

Maternal age at birth. Secondary outcome measures were body mass index, smoking habits, and psychiatric comorbidities.

**Results:**

Among 384 103 nulliparous women and girls aged 12 to 50 years who gave birth between 2007 and 2014 included in the study, 6410 (1.7%) (mean [SD] age, 25.0 [5.5] years) were identified as having ADHD. The remaining 377 693 women and girls without ADHD (mean [SD] age, 28.5 [5.1] years) served as the control group. Teenage deliveries were more common among women and girls with ADHD than among women and girls without ADHD (15.3% vs 2.8%; odds ratio [OR], 6.23 [95% CI, 5.80-6.68]). Compared with women and girls without ADHD, those with ADHD were more likely to present with risk factors for adverse obstetric and perinatal outcomes, including smoking during the third trimester (OR, 6.88 [95% CI, 6.45-7.34]), body mass index less than 18.50 (OR, 1.29 [95% CI, 1.12-1.49]), body mass index more than 40.00 (OR, 2.01 [95% CI, 1.60-2.52]), and alcohol and substance use disorder (OR, 20.25 [95% CI, 18.74-21.88]).

**Conclusions and Relevance:**

This study found that women and girls with ADHD were associated with an increased risk of giving birth as teenagers compared with their unaffected peers. The results suggest that standard of care for women and girls with ADHD should include active efforts to prevent teenage pregnancies.

## Key Points

**Question:**

Is attention-deficit/hyperactivity disorder (ADHD) associated with increased risk of teenage birth?

**Findings:**

This nationwide cohort study of 384 103 women and girls in Sweden who gave birth for the first time between 2007 and 2014, including 6410 women and girls with ADHD, found that teenage deliveries occurred at a significantly higher rate among women and girls with ADHD than among those without ADHD (15.2% vs 2.8%).

**Meaning:**

This study suggests that women and girls with ADHD may have an increased risk of giving birth as teenagers compared with their unaffected peers.

## Teaser

This cohort study examines the association of attention-deficit/hyperactivity disorder (ADHD) with teenage pregnancy among women and girls in Sweden.

## Introduction

Attention-deficit/hyperactivity disorder (ADHD) is a prevalent neurodevelopmental disorder characterized by symptoms of hyperactivity, impulsivity, and inattention, and it is associated with a plethora of adverse health outcomes throughout life.^1,2^ The worldwide prevalence of childhood and adolescent ADHD is estimated to be approximately 5%.^3,4^ Importantly, children with ADHD have 2-fold the likelihood of mortality, 2-fold again among individuals whose symptoms persist into adulthood, which is associated with a significantly reduced estimated life expectancy.^5,6^

Adolescents with ADHD are at increased risk of externalizing and risk-taking behaviors compared with their unaffected peers and more often engage in risky sexual behavior, such as earlier initiation of sexual activity and more sexual partners.^7,8^ Consequently, they are at risk of sexually transmitted diseases and unplanned pregnancies.^9,10^ Most previous studies exploring the risk of teenage pregnancies associated with ADHD have been conducted in clinical settings using self-reported measures. The clinical context may render outcomes vulnerable to low power and with limited generalizability to community populations. Furthermore, studies using self-reported measures may be sensitive to recall bias. However, a 2017 Danish nationwide cohort study^5^ that addressed these limitations found increased likelihood of teenage parenthood among individuals with ADHD compared with those without.

Teenage pregnancies are associated with several long- and short-term adverse outcomes for both parents and children. Young parents are at risk of low educational attainment, single habitation, and use of public assistance.^11,12^ Risks for the children include perinatal morbidity and mortality, low socioeconomic status, and low quality of life.^13,14,15,16^ In Sweden, teenage birth rates have decreased from 15.3% of all births in 1973 to 2.4% in 2014,^17^ one of the lowest rates internationally.^18,19^

To our knowledge, the prevalence of teenage pregnancies in women and girls with ADHD has not been fully explored from a clinical perspective. Given the easy access to counseling and contraception, Sweden represents an ideal setting for investigating teenage births in women and girls with ADHD. Independent of socioeconomic status, all women and girls with ADHD have access to adequate contraception and counseling. Thus, this large-scale epidemiological study was designed to explore the prevalence of birth in young women and teenage girls with ADHD and to address modifiable risk factors associated with adverse obstetric and perinatal outcomes, such as smoking, body mass index (BMI), and substance use disorder in these women and girls.^6^

## Methods

This nationwide cohort study was based on data from 6 Swedish national population-based registries. The personal identity number assigned to every Swedish citizen at birth or immigration facilitated information linkage across registries.^20^ The Swedish National Board of Health and Welfare provided data from the Swedish Medical Birth Register, the Patient Register, the Swedish Prescribed Drug Register, and the Cause of Death Register. Statistics Sweden provided data from the Education Register and the Total Population Register.

The Medical Birth Register includes 98% of all births in Sweden, including prospectively collected clinical variables, demographic data, and information on reproductive history, as well as complications during pregnancy, birth, and the neonatal period.^21^ Information was compiled from the standardized antenatal care records, at the first antenatal visit (conducted in approximately gestational week 9), and in gestational week 32 for smoking habit.^22^ Information on year and month of birth and gestational length at birth was collected from standardized birth records.

The Patient Register includes information on dates of hospital admissions and *International Statistical Classification of Diseases and Related Health Problems, Tenth Revision* (*ICD-10*)^23^ diagnoses, with full national coverage since 1987.^24^ The Patient Register has also covered specialized outpatient visits since 2001. Both registers captured information from 2001 to 2014. The Prescribed Drug Register contains information on drug identity using Anatomical Therapeutic Chemical (ATC) classification codes and dates of prescriptions for the entire Swedish population since July 2005.^25^ The Cause of Death Register provides information on dates of all registered deaths since 1958. The Education Register contains information on highest level of education achieved of all Swedish individuals and the Total Population Register contains information on the identity of all residents born in Sweden since 1932.

This study was approved by the ethical review board in Uppsala, Sweden. Patient data confidentiality was assessed and approved by the National Board of Health and Welfare, Statistics Sweden, and the ethical review board before deidentified data from national registers were disclosed to the research team. The ethical review board waived informed consent because the exposure was within normal clinical practice and informed consent is not feasible in nationwide register-based data linkages. The study was designed, conducted, and reported adhering to the Strengthening the Reporting of Observational Studies in Epidemiology (STROBE) reporting guideline. Data analyses were conducted from October 7, 2018, to February 8, 2019.

### Study Population and Exposure

All Swedish nulliparous women and girls who gave birth between January 1, 2007 and December 31, 2014, were identified through the Medical Birth Register and included in the study (Figure). We identified women and girls who were treated with stimulant or nonstimulant medication for ADHD (ie, ATC classification code N06BA) from July 1, 2005, to December 31, 2014, through the Prescribed Drug Register. Stimulant and nonstimulant medications are indicated specifically for treatment of ADHD.^26^ Furthermore, according to Swedish national clinical guidelines, ADHD should be diagnosed after evaluation of specialized teams of psychiatrists and psychologists, including childhood history and medical and cognitive assessments.^27^ All other women and girls in the cohort served as the control group.

**Figure.**
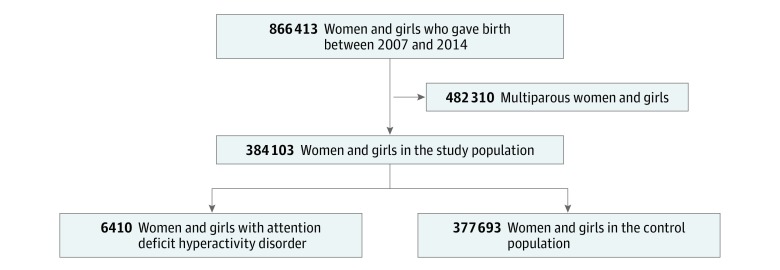
Cohort Recruitment Flowchart

### Outcomes

The Medical Birth Register was used for information on maternal age at birth, height, weight, and smoking habits. Age at birth was categorized as teenagers (women and girls aged <20 years) and nonteenagers (women aged ≥20 years). Body mass index (calculated as weight in kilograms divided by height in meters squared) was calculated based on information on weight and height at first antenatal visit and categorized according to the World Health Organization as less than 18.50, 18.50 to 24.99, 25.00 to 29.99, 30.00 to 34.99, 35.00 to 39.99, and more than 40.00. Information on smoking was captured at the first antenatal visit and in gestational week 32, and ongoing smoking, regardless of quantity, was coded as yes; no smoking was coded as no.

We collected information on psychiatric comorbidities from the Patient Register between January 1, 2001, and December 31, 2014. Women and girls who received a diagnosis according to *ICD-10*^23^ at least twice before the index pregnancy were considered to fulfill diagnostic criteria for comorbid bipolar disorder (*ICD-10* codes F30-F31), schizophrenia or other psychotic disorder (*ICD-10* codes F20-F29), emotionally unstable personality disorder (*ICD-10* code F60.3), or substance use disorders (*ICD-10* codes F10-F16 or F18-F19). As mood and anxiety disorders are mostly diagnosed in primary care, we used dispensed prescription of antidepressant treatment (ATC classification code N06A) from the Prescribed Drug Register as a proxy for indexing past episodes. Furthermore, as substance use disorder may vary over time, we analyzed presence of substance use disorder in 12 months preceding estimated date of conception, and only required 1 diagnostic occasion in this period. At least 1 filled prescription of ADHD treatment (ATC classification code N06BA) in the year prior to the estimated date of conception and during pregnancy were used to define ADHD treatment during the year preceding pregnancy and during pregnancy, respectively.

### Maternal Characteristics

Data on maternal education in 2014 were collected from the Education Register. Maternal education was categorized as less than 10 years completed, 10 to 12 years completed (ie, high school), and more than 12 years completed (ie, college). Data on maternal country of birth were collected from the Total Population Register. Maternal country of birth was classified as Nordic (ie, Denmark, Finland, Iceland, Norway, or Sweden), European, or other.

### Statistical Analysis

Characteristics of the population were described according to exposure with absolute and relative frequencies. Logistic regression models were used to estimate the magnitude of the associations between age at first pregnancy, risk factors for adverse obstetric and perinatal outcomes, psychiatric comorbidities, and ADHD diagnosis, presented as odds ratios (ORs) with 95% CIs. Missing data were excluded. As the emphasis in this study was on the total burden of disease, no adjustments were made. In addition, we refrained from any adjustment on any of the outcomes by ADHD treatment during the year preceding pregnancy, as we would not be able to ascertain whether participants had stopped taking their medication when planning a pregnancy or not.

Furthermore, differences in risks factors for adverse obstetric and perinatal outcomes (ie, smoking, underweight, overweight, obesity, and substance use disorder) were explored in distinct age-at-birth subgroups among women and girls with ADHD (<20 years vs ≥20 years). All analyses were performed using SPSS statistical software version 25.0 (IBM). *P* values were 2-tailed, and statistical significance was set at less than .05.

## Results

The total cohort included 384 103 women and girls, including 6410 women and girls with ADHD (mean [SD] age, 25.0 [5.5] years) and 377 693 women and girls without ADHD (mean [SD] age, 28.5 [5.1] years). The sociodemographic and clinical variables of women and girls in the cohort are presented in Table 1. The overall rate of teenage births in the study was 3.0% (11 615 births). Teenage deliveries were significantly more common among women and girls with ADHD (15.3%) than in women and girls without ADHD (2.8%). Women and girls with ADHD contributed 8.4% to all teenage deliveries during the period. Compared with women and girls without ADHD, those with ADHD were associated with a 6-fold increased risk of giving birth when they were younger than 20 years (OR, 6.23 [95% CI, 5.80-6.68]).

**Table 1.** Association of ADHD With Demographic Characteristics of Nulliparous Women and Girls Who Gave Birth Between 2007 and 2014

Characteristic	Total Cohort, No. (N = 384 103)	Women and Girls, No. (%)		Odds Ratio (95% CI)
		With ADHD (n = 6410)	Without ADHD (n = 377 693)	
Age, y				
<20	11 615	980 (15.3)	10 635 (2.8)	6.23 (5.80-6.68)
≥20	372 484	5430 (84.7)	36 7054 (97.2)	1 [Reference]
Missing	4	0	4 (0)	NA
Country of birth				
Nordic countries^a^	305 196	5977 (93.2)	299 219 (79.2)	1 [Reference]
European	13 433	86 (1.3)	13 347 (3.5)	0.32 (0.26-0.40)
Other	65 474	347 (5.4)	65 127 (17.2)	0.27 (0.24-0.30)
Body mass index^b^				
<18.50	10 838	201 (3.1)	10 637 (2.8)	1.29 (1.12-1.49)
18.50-24.99	225 681	3261 (50.9)	222 420 (58.9)	1 [Reference]
25.00-29.99	84 018	1502 (23.4)	82 516 (21.8)	1.24 (1.17-1.32)
30.00-34.99	25 849	594 (9.3)	25 255 (6.7)	1.60 (1.47-1.75)
35.00-39.99	7926	221 (3.4)	7705 (2.0)	1.96 (1.70-2.25)
>40.00	2729	78 (1.2)	2651 (0.7)	2.01 (1.60-2.52)
Missing	27 062	553 (8.6)	26 509 (7.0)	NA
Education, y				
<10	57 731	3117 (48.6)	54 614 (14.5)	8.74 (8.20-9.32)
10-12	109 602	1839 (28.7)	107 763 (28.5)	2.61 (2.44-2.80)
>12	212 350	1378 (21.5)	210 972 (55.9)	1 [Reference]
Missing	4420	76 (1.2)	4344 (1.2)	NA
Smoking during first trimester				
No	346 160	4445 (69.3)	341 715 (90.5)	1 [Reference]
Yes	22 844	1659 (25.9)	21 185 (5.6)	6.02 (5.68-6.38)
Missing	15 099	306 (4.8)	14 793 (3.9)	NA
Smoking during third trimester				
No	348 320	4498 (70.2)	343 822 (91.0)	1 [Reference]
Yes	15 670	1294 (20.2)	14 376 (3.8)	6.88 (6.45-7.34)
Missing	20 113	618 (9.6)	19 495 (5.2)	NA

Abbreviations: ADHD, attention-deficit/hyperactivity disorder; NA, not applicable.

^a^ Includes Denmark, Finland, Iceland, Norway, and Sweden.

^b^ Calculated as weight in kilograms divided by height in meters squared.

In addition, compared with women and girls without ADHD, women and girls with ADHD were more likely to present with risk factors for adverse obstetric and perinatal outcomes, including being underweight (BMI <18.50: OR, 1.29 [95% CI, 1.12-1.49]), obese (BMI >40.00: OR, 2.01 [95% CI, 1.60-2.52]), or smokers. Smoking during the first trimester was reported by 25.9% of women and girls with ADHD compared with 5.6% of women and girls without ADHD (OR, 6.02 [95% CI, 5.68-6.38]). Furthermore, 20.2% of women and girls with ADHD continued to smoke when entering the third trimester compared with 3.8% of women and girls without ADHD (OR, 6.88 [95% CI, 6.45-7.34). In addition, 48.6% of women and girls with ADHD had 10 years of education or less compared with 14.5% of women and girls without ADHD, and 93.2% of women and girls with ADHD were born in Nordic countries compared with 79.2% of women and girls without ADHD.

Table 2 presents psychiatric comorbidities prior to pregnancy and use of stimulant, nonstimulant, and antidepressant medication during pregnancy. Alcohol and substance use disorder was the most common comorbidity, diagnosed in 14.7% of women and girls with ADHD (OR, 20.25 [95% CI, 18.74-21.88]). During the year preceding pregnancy, 6.6% of women and girls with ADHD received a substance use disorder diagnosis (OR, 27.03 [95% CI, 24.06-30.37]). Compared with women and girls without ADHD, women and girls with ADHD were more likely to have received a diagnosis for bipolar disorder (OR, 17.67 [95% CI, 15.58-20.04]), schizophrenia or other psychotic disorder (OR, 7.92 [95% CI, 6.16-10.17]), or emotionally unstable personality disorder (OR, 22.04 [95% CI, 19.59-24.79]). Prior antidepressant use was more common among women and girls with ADHD than women and girls without ADHD (OR, 8.71 [95% CI, 8.28-9.16]). Overall, 7.6% of women and girls with ADHD continued to use stimulant or nonstimulant ADHD medication during pregnancy, and 16.4% used antidepressants during pregnancy (OR, 7.69 [95% CI, 7.18-8.24]).

**Table 2.** Association of ADHD With Psychiatric Comorbidities, ADHD Treatment, and Antidepressants During Pregnancy in Nulliparous Women and Girls Who Gave Birth Between 2007 and 2014

Variable	Total Cohort, No. (N = 384 103)	Women and Girls, No. (%)		Odds Ratio (95% CI)
		With ADHD (n = 6410)	Without ADHD (n = 377 693)	
Bipolar disorder	1463	325 (5.1)	1138 (0.3)	17.67 (15.58-20.04)
Prior antidepressant treatment	45 574	3352 (52.3)	42 222 (11.2)	8.71 (8.28-9.16)
Emotionally unstable personality disorder	1509	393 (6.1)	1116 (0.3)	22.04 (19.59-24.79)
Alcohol and substance use disorder	4118	940 (14.7)	3178 (0.8)	20.25 (18.74-21.88)
Alcohol and substance use disorder in the year preceding the pregnancy	1418	426 (6.6)	992 (0.3)	27.03 (24.06-30.37)
Schizophrenia or other psychotic disorder	596	70 (1.1)	526 (0.1)	7.92 (6.16-10.17)
ADHD treatment during pregnancy	489	489 (7.6)	0	NC
Antidepressant use during pregnancy	10 462	1053 (16.4)	9409 (2.5)	7.69 (7.18-8.24)

Abbreviations: ADHD, attention-deficit/hyperactivity disorder; NC, not calculated.

### Risk Factors of Adverse Obstetric and Perinatal Outcomes Associated With Age in Women and Girls With ADHD

Table 3 presents risk factors for adverse obstetric and perinatal outcomes associated with age at birth in women and girls with ADHD. Compared with women with ADHD who gave birth when they were 20 years or older, women and girls with ADHD who gave birth when they were younger than 20 years were more likely to smoke during the first trimester (OR, 2.02 [95% CI, 1.75-2.34]) and when entering the third trimester (OR, 2.02 [95% CI, 1.73-2.37]). Being underweight was more common in the teenaged mothers with ADHD than mothers 20 years and older with ADHD (OR, 1.97 [95% CI, 1.42-2.73]). Teenaged mothers with ADHD were less likely to have received a diagnosis of substance use disorder the year prior to pregnancy than women with ADHD who gave birth after age 20 years (OR, 0.72 [95% CI, 0.53-0.98]). They were also more likely to use stimulant or nonstimulant ADHD medication in the year preceding the pregnancy (OR, 1.80 [95% CI, 1.55-2.08]) and were more likely to continue stimulant or nonstimulant treatment during pregnancy (OR, 1.73 [95% CI, 1.38-2.16]).

**Table 3.** Obstetric Risk Factors in Nulliparous Women and Girls With ADHD by Age at Birth

Variable	Women and Girls, No (%)		Odds Ratio (95% CI)
	Aged ≥20 y (n = 5430)	Aged <20 y (n = 980)	
Smoking during the first trimester	1284 (24.8)	375 (40.1)	2.02 (1.75-2.34)
Smoking during the third trimester	1001 (20.3)	293 (34.0)	2.02 (1.73-2.37)
Body mass index^a^			
<18.50	147 (3.0)	54 (6.1)	1.97 (1.42-2.73)
18.50-24.99	2749 (55.2)	512 (58.2)	1 [Reference]
25.00-29.99	1290 (25.9)	212) (24.1)	0.88 (0.74-1.05)
30.00-34.99	523 (10.5)	71 (8.1)	0.73 (0.56-0.95)
35.00-39.99	200 (4.0)	21 (2.4)	0.56 (0.36-0.89)
>40.00	68 (1.4)	10 (1.1)	0.79 (0.40-1.54)
Alcohol or substance use disorder	415 (14.1)	110 (11.2)	1.05 (0.88-1.25)
Alcohol or substance use disorder during 12 mo preceding the pregnancy	157 (5.3)	50 (5.1)	0.72 (0.53-0.98)
ADHD treatment during 12 mo preceding the pregnancy	685 (23.3)	341 (34.8)	1.80 (1.55-2.08)
ADHD treatment during pregnancy	213 (7.2)	112 (11.4)	1.73 (1.38-2.16)

Abbreviation: ADHD, attention-deficit/hyperactivity disorder.

^a^ Calculated as weight in kilograms divided by height in meters squared.

## Discussion

This population-based cohort study examined the association of ADHD with age at first childbirth and associated medical and psychiatric risks. Our results showed an increased likelihood for teenage childbirth in women and girls with ADHD. Pregnant women and girls with ADHD presented with a number of medical and psychiatric comorbidities, among which substance use disorder was the most common.

Although teenage pregnancies are a rare occurrence in women and girls in Sweden with or without ADHD, as suggested by an overall rate of teenage deliveries of 3.0% in this study, women and girls with ADHD were associated with a 6-fold increased risk for teenage birth compared with women and girls without ADHD and contributed 8.4% to all teenage births. This is evident despite the widespread availability of contraception in Sweden. Becoming a mother at such early age is associated with long-term adverse outcomes for both women and their children.^11,12,13,14,15,16^ Consequently, our findings argue for an improvement in the standard of care for women and girls with ADHD, including active efforts to prevent teenage pregnancies and address comorbid medical and psychiatric conditions. In addition, antenatal care should focus on adequate measures to reduce effect of obstetrics risk factors in these women and girls. Unfortunately, owing to a lack of understanding and specific research addressing sex differences, ADHD in women and girls is still underrecognized, misdiagnosed, and, once appropriately diagnosed, suboptimally treated.^28^

Some important but unexplored hypotheses that may explain our results are that women and girls with ADHD receive inadequate contraceptive counseling, inadequately respond to counseling, fail to access or act on counseling, or experience more adverse effects from hormonal contraceptives. As to contraceptive counseling, Swedish youth clinics have made counseling and contraception easy to access at low cost for this population. However, collaboration between psychiatric care clinics for youths and specialized youth clinics is needed to specifically address and provide adequate care, including contraception, for women and girls with ADHD. Thus, it is possible that a lack of transdisciplinary knowledge and a subsequent team effort failure may be associated with the high proportion of teenage births among women and girls with ADHD. However, even if provided with such assistance, women and girls with ADHD may be less likely to act on or implement such counseling owing to their disorder and its associated deficits in self-regulation.

Adverse mental health effects from hormonal contraceptives are increasingly reported by young users,^29^ and an increasing proportion of younger women and girls abstain from hormonal contraceptives owing to a fear of future adverse effects.^30^ Previous placebo-controlled randomized clinical trials have suggested minor mood disturbances with combined hormonal contraceptive use in healthy women,^31,32,33^ especially among women with a history of mental health conditions.^34^ As women with chronic psychiatric conditions are excluded from most clinical trials on hormonal contraceptives, these studies provide limited guidance in selecting contraceptives most suitable for this group. Further studies on contraceptives in women and girls with ADHD are needed.

Our study found that young women and girls with ADHD were more likely to present with a number of risk factors for adverse obstetric and perinatal outcomes, such as underweight and smoking. Our findings suggest that delaying first childbirth until after age 20 years may be advantageous for women with ADHD in terms of the risks associated with smoking and underweight for the offspring. This is in line with the current conceptualization of ADHD as a neurodevelopmental disorder with delayed brain maturation associated with age-inappropriate symptoms of disinhibiting, risk taking, and impulsivity.^35^ However, while our findings may imply that the obstetric risk factor profile is somewhat normalized with increased age at first birth, this may also be due to reverse causation, ie, women and girls with ADHD with a lower symptom load and high functioning may simply choose to delay their first birth.

Pharmacological ADHD treatment in the year preceding the first pregnancy was not frequent in any of the age groups. Stimulant drug treatment has been associated with a decrease of core symptoms and adverse outcomes associated with ADHD.^36^ Indeed, studies controlling for confounding by indication by using individuals as their own comparisons during periods with and without treatment suggest that stimulant medication may reduce or even ameliorate relevant adverse outcomes, such as educational failure,^37,38^ and decrease risk for unwanted outcomes associated with ADHD, such as substance use disorder,^39^ motor vehicle crashes,^40^ suicidal behavior,^41^ and criminal behavior.^42^ While it may be hypothesized that not using stimulant medication is associated with increased risk of unplanned pregnancy, this study cannot confirm any such association. Low rates of ADHD drug treatment may simply be owing to some women discontinuing psychotropic medication when planning for pregnancy.

### Limitations

Our study has limitations. The ascertainment of ADHD diagnosis was based on prescribed medication unique for the treatment of ADHD rather than *ICD-10* or *Diagnostic and Statistical Manual of Mental Disorders*^43^ diagnoses. However, Swedish national guidelines state that medication should be reserved for ADHD treatment when other supportive interventions have failed,^27^ indicating that our proxies most likely underestimated the incidence of ADHD and identified the most severe cases. Thus, while our definition of exposure probably could not avoid false-negatives, we considered bias due to false-positives more unlikely. Also, it should be noted that our findings did not rule out the possibility of an association of age at first pregnancy with more refined ADHD-related neurocognitive deficits. To further explore this, studies using more detailed measures of ADHD symptom severity are warranted. Additionally, as in all observational studies, we could not fully rule out selection bias due to a lack of intact information on exposure and outcome variables.

## Conclusions

In conclusion, our data replicated the findings from a 2017 Danish cohort study by Ostergaard et al^5^ suggesting increased likelihood of teenage parenthood among women and girls with ADHD by showing that women and girls with ADHD were associated with a 6-fold increased risk of giving birth as teenagers compared with their unaffected peers. Furthermore, our results expanded the findings from the prior Danish study^5^ by suggesting that women and girls with ADHD are a significant and underrecognized group with several obstetric risk factors and comorbid medical and mental health concerns, among which substance use disorder was the most common. Standard of care in women and girls with ADHD should include active efforts to prevent teenage pregnancies to reduce long-term adverse consequences for both mothers and children. Transdisciplinary collaboration between psychiatric clinics for youths and specialized youth clinics, as well as further studies on tolerability of hormonal contraception in women and girls with ADHD, are warranted to provide adequate care and suitable contraception for youth with neurodevelopmental disorders.
